# Bromide of Ammonium as an Anodyne

**Published:** 1869-08

**Authors:** C. N. Cooper

**Affiliations:** Cresco, Iowa


ARTICLE XXXII.

### BROMIDE OF AMMONIUM AS AN ANODYNE.

By C. N. COOPER, M.D., Cresco, Iowa.

Mrs. B., aged 28, gave birth to her first child on the morning of July 6th, after a tedious labor. Her attending physician ordered salts to move her bowels. Salts were given, but with no effect. Friday evening, Dr. C., of this place, was called, and requested me to see the case with him. Patient complained of severe pain in epigastrium. Dr. C. gave Dov. pul. freely, with no effect. He then tried morph. and chloroform, but she became worse. By his request I injected morph. gr. $\frac{1}{4}$ into the arm, which quieted her at once, but the relief was only temporary. A free enema gave relief, which it was hoped would be permanent. Oil was administered on the following morning. Opiates were ordered to be given freely if the pain returned. At noon her distress was very great, and the opiates had no effect. I was called at once. She was now not only suffering the pain spoken of, but at nearly every breath there was spasmodic contraction of the diaphragm and abdominal muscles over the epigastrium. I gave morph. gr. $\frac{1}{6}$, br. ammo. gr. viij, and spr. ammo. ar. gtts. xv. In a few minutes she said she felt different, but still some pain. I ordered the same to be continued every half hour till relief, which followed the fourth dose. I visited her in the evening and found her comfortable and happy. July 11th, patient doing well, no pains, except slight discomfort, shifting back and forth between stomach and uterus. She has had no more trouble. I cannot say whether the relief was due to the bromide alone, or its combined action with the morphine. My opinion is that the bromide relived *nervous irritation* and *assisted* the morphine in relieving the pain.

